# London parochial burial records from 1563 to 1665 indicate higher plague death rates for males than females: Some possible demographic and social explanations

**DOI:** 10.1371/journal.pone.0272278

**Published:** 2022-08-05

**Authors:** Xavier Didelot, Charles Morris Evans

**Affiliations:** 1 School of Life Sciences and Department of Statistics, University of Warwick, Coventry, United Kingdom; 2 Department of History, School of History & Cultures, University of Birmingham, Birmingham, United Kingdom; Hebrew University, ISRAEL

## Abstract

The burial rates of males and females in early modern central London were compared to investigate a possible bias towards male mortality in the plague years of 1563, 1593, 1603, 1625 and 1665. The burial records of sixteen parishes were examined and compared with the five-year periods immediately preceding each plague year when recorded burials were substantially less. A markedly higher burial rate for males was detected in each plague year but this can be partly attributed to a general preponderance of males in the central London population since there was a similar but lesser bias in non-plague years. In the plague years the difference between the frequency of male and female adult burials appears to have been enhanced by the preferential migration of women of childbearing age out of the city since fewer births were recorded in months when plague was rife. Furthermore, when a sample of households was investigated, husbands were significantly more likely to have been buried than their wives. These findings were largely applicable to the plague years of 1603, 1625 and 1665 but were far less apparent in 1563 and 1593. In general, there were more burials of boys than girls in non-plague years which is the expected consequence of their greater vulnerability to childhood diseases. This difference diminished in plague years so that the burials of girls and boys approached parity at a time when burials of children of both sexes were significantly increased. Possibly, plague did not discriminate between the sexes and this characteristic tended to mask the usual vulnerability of boys.

## Introduction

A recent study of the burial register from the central London parish of St Peter, Cornhill revealed that male inhumations exceeded those of females by 1.54 to 1 and 1.69 to 1 respectively for the plague years of 1593 and 1603 thus suggesting a higher plague mortality for males [1]. This striking observation is not exceptional for London parishes at this time since an earlier investigation of the parish records of St Botolph’s-without-Bishopsgate found that 1.46 men were buried for each woman in 1603 whilst the ratio was 1.37 to 1 in the plague year of 1625 [2]. This trend was confirmed in a wider ranging study by Roger Finlay, incorporating six central London parishes, which produced a similar result for 1603 but indicating that this ‘mysterious’ phenomenon was not repeated in 1593 or 1625 [3]. Finlay noted a wide range of sex ratios between parishes and commented that ‘the study of differential plague mortality between males and females is… exceptionally difficult and little sense can be made of it…’ at the same time calling for more research to investigate the problem.

The actual reason for this implied bias towards male deaths in London is yet to be explained and is by no means typical of the ratios observed in studies of medieval and early modern plague epidemics elsewhere. Ell, for example, found that many more females than males died in Venice during the plague epidemic of 1630 [4] but Lazzari *et al* could find no difference in burial frequency between the two sexes in that city [5]. A preponderance of female deaths in the province of Parma and the city of Carmagnola (northern Italy) in 1630 has been reported but not in the city of Nonantola where male deaths exceeded that of females [6]. Elsewhere, a sex ratio of 1.37 females to 1 male was found in a sample of 606 deaths in Penrith (Cumbria) in 1597/8 [7], whereas a sex ratio of 1.09 males to 1 female was found in the village of Eyam (Derbyshire) in the outbreak of 1665/6 [8]. Variations in the mortality ratio between the sexes can also take on a more nuanced character; for instance, in a study of plague epidemics in the Southern Netherlands from 1349 to 1450 an excess of male burials over female burials was found in non-plague years but, in plague years, female burials exceeded those of males leading to the conclusion that women died in greater proportions during plagues compared to normal times [9].

Investigations of skeletal material, reliably associated with plague epidemics, have not resolved this problem. For instance, a study of the East Smithfield Black Death cemetery in London found that gender did not significantly affect the risk of mortality [10]. On the other hand, a sample of 503 skeletons unearthed in the New Churchyard (N.E. London), used for plague burials in outbreaks from 1620 onwards, showed a sex ratio of 1.3 males to 1 female although burial register evidence suggested a smaller ratio of 1.1 males to 1 female [11]. Margerison and Knüsal [12] compared burials in the Royal Mint site, London (a Black Death cemetery dated 1349) with the St Helen-on-the-Walls, York cemetery which was in use from the late 12^th^ century to 1550. The former was said to represent a ‘catastrophic’ assemblage and the latter represented an ‘attritional’ type. The former exhibited a sex ratio of 1.27 males to 1 female for those skeletons whose sex could be ascertained, whereas the latter showed a ratio of 0.89 males to 1 female. On the other hand, Castex and Kacki could not detect a preferential mortality in 14^th^ and 16^th^ century in four plague burial sites in Europe [13].

A useful starting point of reference for the investigation of sex differences in burial frequency is that the ratio of male to female births, in the absence of intervention, is generally 105–107 males born per 100 females [14]. This phenomenon was first noted in 1662 by John Graunt for the population of London, confirmed statistically by John Arbuthnot in 1710 and has since been endorsed by many later studies of human populations [15]. Thus, if plague kills indiscriminately, we might yet expect a modest excess of male deaths. However, males are generally more susceptible to a variety of infectious and degenerative diseases than females and this is particularly true in childhood [16, 17]. Early modern London seems to reflect this trend as Finlay [18] found that the death rate of children up to the age of 15 was greater for boys than girls but since four parishes only were investigated the general applicability of this finding is limited.

It would be helpful to be able to understand what factors conspire to disturb the expected ratios in times of plague and why their impact seems to vary so much according to time and place. This study has therefore been carried out to investigate sex differences in mortality in London plague epidemics using the burial records of sixteen central parishes over a series of five plague years between 1563 and 1665 to provide a broad base from which to draw conclusions. The burial data is compared with statistics compiled for the five non-plague years preceding each plague year which should indicate if differential sex ratios reflect an ongoing demographic phenomenon or, alternatively, an acute response to a plague epidemic.

It is important to note that the City of London and surrounding areas underwent significant growth in population over the period covered by this paper. It is well established that the bulk of this growth took place in the suburbs and that the mortality statistics reflect this [19]. Paul Slack found that the trend in epidemic mortality due to plague differed across the wider metropolis [20] and that for the central parishes, the epidemic of 1563 was the most severe followed by that of 1603 but the epidemics of 1593, 1625 and 1665 were less severe. Northern, southern and western suburbs, on the other hand, were hardest hit in 1625 and 1665. Other work has confirmed this trend showing that by 1665 the plague mortality in the central intra-mural parishes was considerably lower than elsewhere in the metropolis [21]. Our paper focuses on the wealthier central parishes and whilst there are clearly some advantages in studying a wider area as demonstrated by Cummins *et al* [21] there is greater potential for more detailed studies in the smaller central parishes since many have burial registers which provide sufficient detail to enable the sub-division of the population into ‘children’, ‘servants’ and ‘adults’ [1]. We are then able to find out if plague influences the burial sex ratio in a similar way in each demographic grouping.

One interesting phenomenon, which may be helpful for this study of burial sex ratios, was identified by John Graunt, a contemporary observer of the plague in London from 1603 onwards, who noted that the birth rate reduced at the height of the plague in 1603. He speculated that one of the contributing factors was the flight of pregnant women since this could contribute to a higher ratio of adult male to female burials if the latter either escaped the epidemic or died elsewhere [22]. The number of births recorded in plague years, within our sample of central London parishes is therefore compared to non-plague years to see if Graunt’s observation can be replicated. Possibly, this phenomenon may be connected to the general tendency for flight by the wealthier classes out of London in time of plague [23] but to support this latter hypothesis, it would be helpful if we are able to produce evidence that more females than males left the city leaving males to be over-represented in the burial registers. Up to the present time, there appears to have been no systematic investigation of this possibility reported in the literature although there is some anecdotal evidence to suggest that it may be true. For example, it is said that magistrates and those in public office would send their wives and children away but remain behind to fulfil their duties [24]. Generally, contemporaneous writers admit that flight was a sensible course of action but there was also an understanding that magistrates, clergy and physicians were morally obliged to stay, thus many writers simply focused on the condemnation of the rich who, on fleeing, left the poor behind to suffer in a disorganised city [25]. Even so, these authors do not comment on the possibility that women were more likely to flee than men. A sample of households will therefore be examined to see if there is any discernible difference in the rate of burials of husband and wives. If, indeed, it could be shown that more husbands than wives died within households it would be additional evidence to suggest that more women fled than did their partners thus contributing towards the bias towards adult males.

## Materials and methods

### Parochial registers

As a source of data, we make use of the parochial registers transcribed and published by the Harleian Society as others have done previously [1, 3, 26]. In addition, the register of the parish of St Christopher le Stocks, which was privately published, was also utilised. Some London parish registers date back to 1538 and others to the accession of Queen Elizabeth in 1558 but it must be stressed that they vary in terms of the information recorded so that their usefulness as data sources for epidemiological study varies across parishes and over time [27]. These publications generally present lists of baptisms, marriages and burials but there is plainly no uniform standard for compiling records. For instance, the St Peter, Cornhill register is only one of three to record age at death over an extended period [2] but it did not record cause of death in the plague year 1603, whereas the register of the adjacent St Michael, Cornhill did not record age at death but consistently attributed deaths to plague over this same period [1]. Another example of the difficulties encountered is found in the burial register of St Olave, Hart Street which, commencing on 26^th^ April 1563, began by giving details of occupation, family status (‘son of’, ‘wife of’ etc) but from 28^th^ August recorded the names of the deceased only. This change coincided with the burial of the parish clerk and the register did not return to recording full details until the appointment of a new clerk on the 7^th^ October. Nevertheless, as a minimum, all registers inspected give the date of burial and the first and family name of deceased which is sufficient to compute the numbers of burials in each year. The gender of an individual can generally be determined by examining the first name of an individual but in exceptional cases difficulties arise because a name may be used for individuals of both sexes. A case in point is that of Frances spelt variously: Fraunces, Francis or Frauncis. Nevertheless, this difficulty can usually be overcome by accompanying information identifying the deceased as ‘son of’, ‘daughter of’, ‘wife of’ etc.

### Parishes selected for analysis

**Fig 1:**
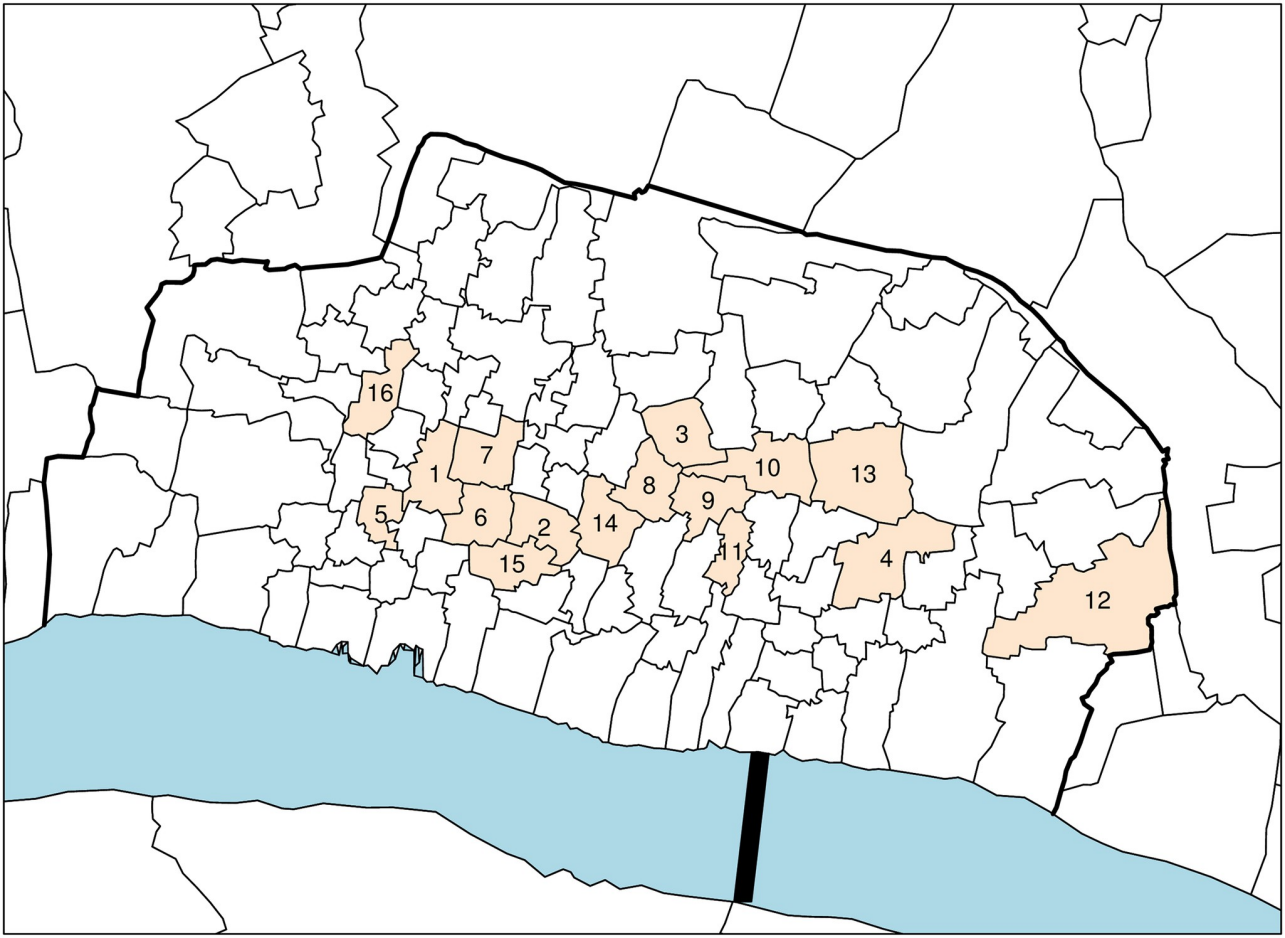
London ‘within-the walls’ to illustrate the location of the parishes selected for investigation. 1. All Hallows, Bread Street; 2. St. Antholin, Budge Row; 3. St. Christopher le Stocks; 4. St. Dionis Backchurch; 5. St. Margaret Moses; 6. St. Mary Aldermary; 7. St. Mary le Bowe; 8. St. Mary Woolnoth; 9 St. Mary Woolchurch Haw; 10. St. Michael, Cornhill; 11. St. Nicholas Acons; 12. St. Olave, Hart Street; 13. St. Peter, Cornhill; 14. St. Stephen, Walbrook; 15. St. Thomas the Apostle; 16. St. Vedast, Foster Lane.

The location of the sixteen named parishes within central London is illustrated on a map (Fig 1). The main criteria for including a register in the study was that it should provide burial data in each of the plague years 1563, 1593, 1603, 1625 and 1665. It was also intended that whenever possible, data from the preceding five non-plague years would also be recorded for purposes of comparison with plague years. However, for several parishes, burial records do not commence until the accession of Queen Elizabeth I in 1558 so for these registers there was little to be done other than follow Roger Finlay and count the burials recorded for the prior four years from 1559 to 1562 inclusive [3]. Only a few of the burial registers we investigated recorded the cause of death so our statistics necessarily focus on the total number of burials in each year observed.

### Categorisation of the burial data

Children were identified from the text as ‘son of’ or ‘daughter of’ the head of household. In a study of the parish of St Peter, Cornhill where ages at burials were recorded, it was found that, almost without exception, the ages of individuals recorded as ‘children’ were between 0 and 19 years [1]. Stillborn children were not included in this study and it was rare, indeed, for the gender to be recorded in such cases. Servants were identified by the designations: ‘servant’, ‘maid’, ‘his man’, ‘wench’ and ‘prentice’ often, but not exclusively, with reference to the head of household. There does not seem to be a consistent usage which would have differentiated between domestic servants and apprentices with only a minority of parishes specifically noting the burials of apprentices. Of these, only a small percentage were female. This is despite estimates of the proportion of adult males in early modern London who had been trade apprentices ranging between 10% and 40% within the city walls and some estimates of the proportion of apprentices to adult males within London in 1600 ranging between 19.4 and 24.3% [28, 29]. Thus, it is not possible, using parish records to estimate the relative contribution of domestic servants and apprentices to the category ‘servants’. The age range of ‘servants’ was found to be between 15 and 25 in the parish of St Peter, Cornhill [1] and thus this grouping provides a useful cohort for study which is intermediate between that of children and adults.

The category ‘adults’ included all wives and widows together with men having a recorded occupation. However, it also included a minority of cases where the information recorded was limited to the first and family names of the deceased, some that were designated: ‘brother’, ‘sister’, ‘son-in-law’ or ‘cousin’ and a few others who could be identified as lodgers by the appellation ‘out of’ or ‘from’ followed by a reference to the dwelling of another person who was not associated by family tie.

Unfortunately, some parish records were not suitable for consideration at this level of detail because in some years they consistently failed to record any detail save for the name of the deceased and the date of burial. These parishes were discarded from the analysis in those years. Table 1 lists the parishes included in the study and indicates the total number of burials for each year together with the number discarded due to the parish record being found to be unsuitable. The percentage burials discarded (37.8%) was far greater in 1563 than any other plague year. Nearly 60% of this loss is due to the deficient records of St Peter, Cornhill and St Michael, Cornhill from which 352 burials were discarded. In the case of St. Michael, the burials records of previous years and the first few burials in 1563 give useful detail of the deceased but none thereafter, leading one to suppose that the parish clerk gave up as the number of burials rapidly increased. In the case of St. Michael, Cornhill the original register is lost to us but a transcript from these earlier years were made by a parish clerk who died in 1605 [1].

**Table 1:** Parishes selected for the detailed analysis of ‘adults’, ‘servants’ and ‘children’. The total number of burials for the sixteen parishes in each plague year and thus the percentage number of burials discarded for this analysis are also presented.

	Plague Year					
Parish	1563	1593	1603	1625	1665	Total
All Hallows, Bread Street	✔	✔	✔	✔	✔	
St Antholin, Budge Row	✔	✔	✔	✔		
St Christopher le Stocks	✔	✔		✔	✔	
St Dionis Backchurch	✔	✔	✔	✔	✔	
St Margaret Moses	✔	✔	✔	✔	✔	
St Mary Aldermary	✔	✔	✔	✔	✔	
St Mary le Bowe	✔		✔	✔	✔	
St Mary Woolchurch Haw				✔		
St Mary Woolnoth	✔	✔	✔	✔	✔	
St Michael Cornhill		✔	✔	✔	✔	
St Nicholas Acons	✔	✔	✔	✔	✔	
St Olave, Heart Street	✔	✔	✔	✔	✔	
St Peter, Cornhill		✔	✔	✔	✔	
St Stephen, Walbrook			✔	✔	✔	
St Thomas the Apostle	✔	✔	✔	✔	✔	
St Vedast, Foster Lane		✔	✔	✔		
Burials sixteen parishes	1596	944	1376	1412	1357	6685
Burials selected parishes	993	849	1262	1271	1103	5478
Percentage burials discarded	37.8	10.1	8.3	10.0	18.7	18.1

### Data on birth rates

Records of baptisms were available for fifteen of the sixteen parishes selected for our study—the exception being that of St Vedast, Foster Lane for which no record was available. The monthly tallies of baptisms in these parishes were computed for each of the five plague years under investigation as well as a monthly tally for the same parishes for the year immediately preceding each plague year for the purpose of comparison.

### Household data

To investigate the burial ratio of husband to wives, households were identified and sampled on the basis that two or more individuals, for whom burials were recorded, could be connected by a familial or servant/master relationship. This sampling technique was applied to all parishes where the records were sufficiently detailed but, as explained previously, the quality of records varied between parishes and between years for individual parishes and so some records could not be used for this part of our investigation (see Table 1). It is recognised that definitions of family and household can both be complex and interchangeable but the purpose here is simply to identify a sample of households which may be investigated for the sex ratio of deaths in plague years rather than to make any inference on the general status and composition of households.

## Results

### Recorded burials of male and female subjects in plague and non-plague years

The data recorded to compare sex ratios at burial included a total of 13,068 burials. Typically, each plague year followed the same pattern with a wave of burials concentrated in the late summer and autumn as noted in other studies [21]. As previously reported by Finlay [3], there was substantial variation between parishes during the five plague years. Some parishes, such as St Antholin and St Margaret Moses’ showed considerable dissimilarity between plague years whilst in any given plague year ratios in the 16 parishes could vary between less than 100 and more than 200 male burials per 100 females. The reason for this is not immediately obvious but possible contributing factors might include small samples in some parishes, poor record-keeping by parish clerks and different demographic profiles of parishes. Data from all sixteen parishes were pooled for analysis and the resulting total male and female burials in both plague and non-plague years are presented in Table 2. Again, this data provides evidence of high male to female sex ratios in all the plague years but less so in 1665. However, to prove that plague affected one sex more than the other, it is important to compare plague years with non-plague years. The statistic chosen for this analysis was Pearson’s chi-square test. The null hypothesis (H_0_) generally adopted was that there is no difference between the sexes in terms of burial rates and a p-value < 0.05 was used as the level of statistical significance suggesting a rejection of the null hypothesis. A supplementary analysis, based on the calculation of 95% confidence intervals, was also performed for all comparisons to provide a separate indication of statistical significance when the intervals did not overlap.

**Table 2:** A comparison between the frequency of all male and female burials for five plague years and the preceding non-plague years in sixteen parishes. The combined totals for plague and non-plague years are also presented.

		1563	1593	1603	1625	1665	Total
**Plague years**	Male	938	554	806	803	667	3768
	Female	677	365	549	608	601	2800
	Sex ratio	1.386	1.518	1.468	1.321	1.110	1.346
	CI	± 0.098	± 0.131	± 0.108	± 0.105	± 0.110	± 0.049
**Preceding non-plague years**	Male	598	626	623	854	933	3634
	Female	431	477	483	699	776	2866
	Sex ratio	1.387	1.312	1.290	1.222	1.202	1.268
	CI	± 0.123	± 0.119	± 0.118	± 0.099	± 0.095	± 0.049

The analysis shows that the average male to female burial ratio for the five plague years was 1.35 males per female but even in non-plague years this ratio was 1.27 to 1. In terms of frequency of male and female burials, the difference between combined totals for plague and non-plague years were not statistically significant (χ^2^ = 1.51, p = 0.219). Next, the total number of male and female ‘servant’ burials during plague years was compared with non-plague years and the results are presented in Table 3. The ratios of male to female servant burials were 1.98: 1 in plague years and 1.94: 1 in non-plague years. This difference between plague and non-plague years was not statistically significant (χ^2^ = 0.082, p = 0.775). The sex ratio remained relatively constant except for a decrease in 1665 but this apparent difference did not reach statistical significance. In both plague and non-plague years, burials of male servants were roughly double that observed for females.

**Table 3:** Servants: A comparison between the frequency of male and female burials for the plague years under study and the preceding, non-plague, five-year periods. The combined totals are also presented.

		1563	1593	1603	1625	1665	Total
**Plague years**	Male	202	134	221	144	94	795
	Female	98	62	105	74	62	401
	Sex ratio	2.061	2.161	2.105	1.946	1.516	1.983
	CI	± 0.240	± 0.300	± 0.231	± 0.279	± 0.319	± 0.119
**Preceding non-plague years**	Male	46	62	63	102	88	361
	Female	23	32	32	52	47	186
	Sex ratio	2.000	1.938	1.969	1.962	1.872	1.941
	CI	± 0.498	± 0.424	± 0.423	± 0.332	± 0.352	± 0.176

The total number of male and female burials of ‘adults’ during plague years was compared with non-plague years and the results are presented in Table 4. Overall, the sex ratios were 1.40 male for each female adult burials in plague years and 1.18 male adult burials for each female burials in non-plague years. This difference between plague and non-plague years was statistically significant (χ^2^ = 8.123; p < 0.005). Also, there was no overlap of the confidence intervals calculated for the total plague and non-plague years thus supporting the likelihood of there being a real difference between plague and non-plague years. This increase did not appear to be wholly consistent across outbreaks since the bias towards males appeared lower for the plague years of 1563 and 1665. Furthermore, the male-to-female burial sex ratio was markedly increased in the plague year of 1603 compared to the previous five non plague years (χ^2^ = 17.343; p < 10^−3^) with ratios of 1.88 to 1 and 1.06. to 1 respectively. Again, there was no overlap of the confidence intervals. This result seems to corroborate Finlay’s earlier observation that the plague year of 1603 appeared be exceptional in some way.

**Table 4:** A comparison between the frequency of male and female ‘adult’ burials for five plague years and the preceding non-plague years in selected parishes. The combined totals for plague and non-plague years are also presented.

		1563	1593	1603	1625	1665	Total
**Plague years**	Male	193	197	268	306	220	1184
	Female	148	123	133	219	208	831
	Sex ratio	1.304	1.602	2.015	1.397	1.058	1.425
	CI	± 0.213	± 0.024	± 0.207	± 0.173	± 0.189	± 0.088
**Preceding non-plague years**	Male	159	293	236	297	323	1308
	Female	94	233	233	268	291	1119
	Sex ratio	1.691	1.258	1.013	1.108	1.110	1.169
	CI	± 0.254	± 0.171	± 0.180	± 0.164	± 0.158	± 0.079
				χ^2^ = 23.514			χ^2^ = 10.387
				p < 10^−3^			p < 0.002

Next, the total number of burials of boys and girls during plague years were compared with non-plague years and the results are presented in Table 5. The ratios of male to female child burials were 1.26 to 1 in non-plague years but 1.04 to 1 in plague years meaning that in plague years the excess ratio of male to female burials was substantially reduced. Unexpectedly, this trend was in the opposite direction of change found for adults but was statistically significant (χ^2^ = 10.502, p < 0.002) and appeared to be consistent for all five plague years investigated with 1603 also exhibiting a significant difference (χ^2^ = 5.913, p < 0.02). These statistical findings were also corroborated by lack of overlap of confidence intervals.

**Table 5:** A comparison between the frequency of burials of male and female children for five plague years and the preceding non-plague years in selected parishes. The combined totals for plague and non-plague years are also presented.

		1563	1593	1603	1625	1665	Total
**Plague years**	Male	190	174	254	273	232	1123
	Female	181	159	260	256	215	1071
	Sex ratio	1.050	1.094	0.977	1.066	1.079	1.049
	CI	± 0.203	± 0.214	± 0.172	± 0.170	± 0.185	± 0.083
**Preceding non-plague years**	Male	134	225	230	399	432	1420
	Female	102	174	170	325	364	1135
	Sex ratio	1.050	1.094	0.977	1.066	1.079	1.049
	CI	± 0.256	± 0.197	± 0.197	± 0.146	± 0.139	± 0.078
				χ2 = 5.582			χ2 = 8.979
				p < 0.02			p < 0.005
	CMR	6.71	4.19	6.48	3.65	2.91	

CMRs were calculated by comparing the total child burials in each plague year with the average burials over the previous five years. The CMR for 1563 is adjusted to allow for the different numbers of pre-plague years available for each parish (see method section).

This finding may point to why, when the three social categories ‘adults’, ‘servants’ and ‘children’ were lumped together, no statistically significant trend in the general male to female ratio on account of plague could be identified since the trends observed in adults and children were in opposite directions. A supplementary calculation was made of the total number of children buried in each plague year and this statistic was compared to the average of the previous five non-plague years. The calculation for 1563 was adjusted to take account of the fewer pre-plague yearly counts available because some registers did not commence until 1558. The ratios thus calculated ranged from 2.91 in 1665 to 6.71 in 1563 demonstrating the severe impact of plague on children.

### Birth rates in plague and non-plague years

**Fig 2:**
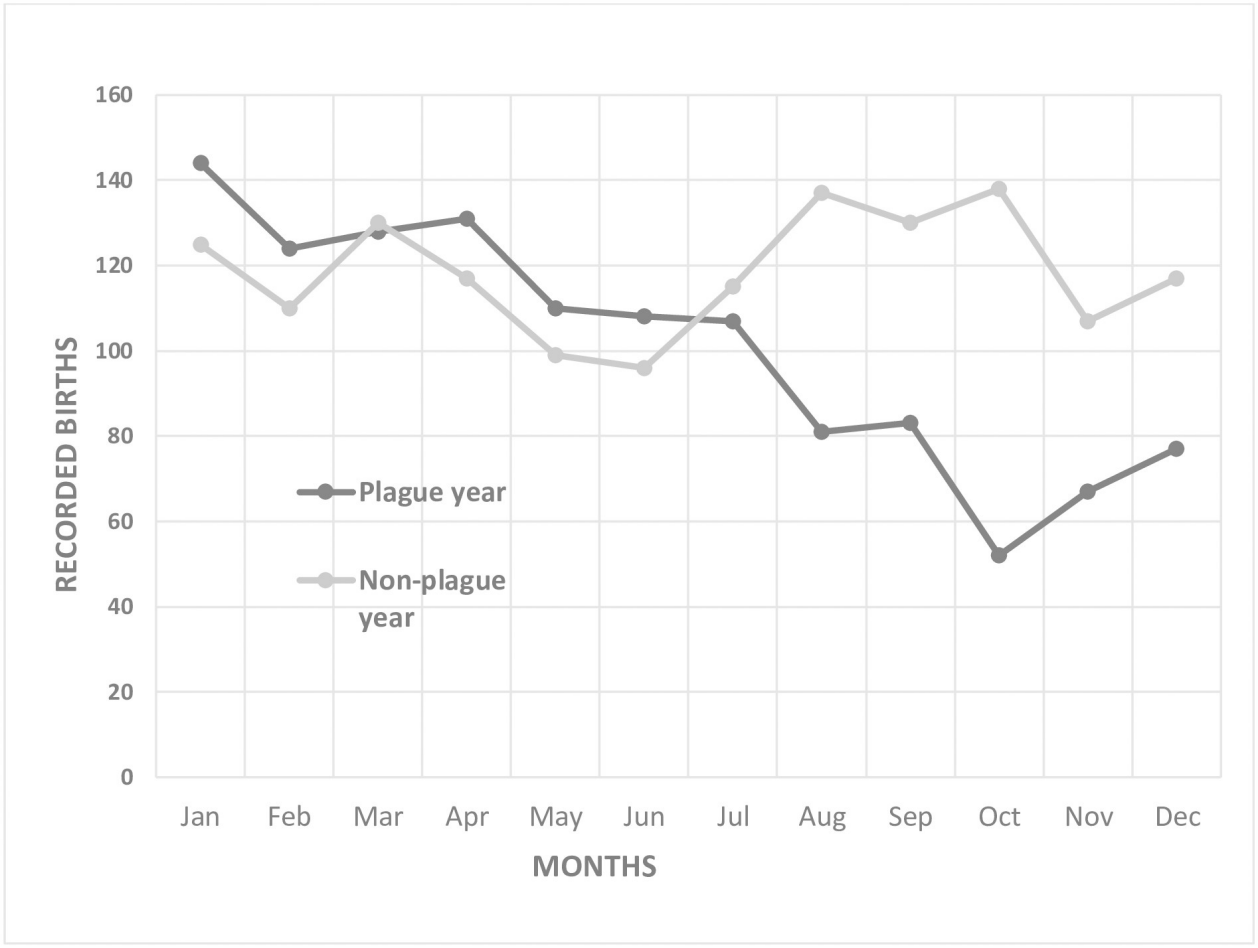
Total of monthly recorded births in sixteen central London parishes in five plague years compared to the monthly recorded births for the five years immediately preceding plague years.

The results for the five plague years were combined and together with the preceding non-plague years are presented in the form of a graph showing the average number of burials in each calendar month (Fig 2). There is a clear indication of a reduction of recorded baptisms in the second half of plague years compared to non-plague years where the number remains relatively stable. To test for significance, baptisms in the first six months of plague and non-plague years were compared with those recorded in the second six-month period for plague and non-plague years using Pearson’s chi-square test. There was a statistically significant difference between plague and non-plague year baptisms (χ^2^ = 50.339, p < 10^−3^) with no overlap of the confidence intervals and was characterised by a reduction in the latter half of plague years.

This finding was examined in more detail by comparing the number of recorded baptisms in the first and second six-month periods for each individual plague year investigated with those recorded in the non-plague years immediately preceding them. The results are presented in Table 6. There was no significant difference in recorded baptisms between plague and preceding non-plague years for 1563 and 1593 but the differences between plague and non-plague years were significant for 1603, 1625 and 1665 with no overlap of confidence intervals. The burial records for 1602 and 1603 were also examined to determine if the number of still births and ‘chrisoms’ (live births of children which died before baptism) could be significant factor. However, in our sample there were only 16 of these in 1602 and 23 in 1603, indicating that these relatively rare events played only a small part in the overall statistics for that plague-year.

**Table 6:** Baptisms recorded in the first and second halves of plague years compared to those recorded in the non-plague year immediately preceding them. Plague years are indicated by the shaded columns.

Years	1562	1563	1592	1593	1602	1603	1624	1625	1664	1665
**1–6 Months**	106	109	145	134	105	154	154	172	167	176
**7–12 Months**	129	106	124	99	131	84	166	85	194	93
χ2	1.407		0.658		19.537		20.499		22.832	
	p = 0.236		p = 0.417		p < 10^−3^		p < 10^−3^		p < 10^−3^	

Chi^2^ values for each 2 x 2 comparison are also presented.

### The ratio of burials of husbands and wives in sampled households

A total of 841 households, spread over the five plague years, were identified by our sampling method. The deaths of husbands and wives were recorded for each household in each of the five plague years and the results are presented in Table 7. The proportion of households registering the death of a husband was compared to that registering the death of a wife for each year, and for all years combined, using a two proportion Z test on a two-tailed basis. We can see that, taken overall, the sex ratio of deaths was 1.42 husbands to one wife which, based on a relatively large sample size, was significantly greater than an expected ratio of 1:1. This finding, albeit based on smaller sample sizes, was replicated in the plague years of 1603 and 1625 but not in 1563, 1593 or 1665. The results parallel those presented earlier for adult males and females although, in this case, the result is separated out from a confounding general preponderance of males in the population. Possibly, the better-off wives might be in a better position to remove themselves from central London but we could not confirm this hypothesis by relating our data with commonly used wealth indicators such as the number of hearths per household or the number of substantial households per parish which are commonly used for this purpose [21, 32]. Also, only 36% of households sampled listed the occupation of the head of household and many registers did not record this information at all. Thus, we were not able to investigate the possibility that the likelihood of flight was related to occupation.

**Table 7:** Proportion of household groups registering the burial of a husband compared to that registering the burial of a wife for five plague years.

Plague Years	1563	1593	1603	1625	1665	Total
Number of households	177	139	183	174	168	841
Burial of husband	45	32	72	83	85	317
Burial of wife	32	20	39	53	79	223
Burial sex ratio	1.41	1.60	1.85	1.57	1.08	1.42
Z score	1.675	1.846	3.752	3.296	0.655	4.768
	N.S	N.S.	p < 10^−3^	p < 10^−3^	N.S.	p < 10^−3^

The sex ratios for each year, combined totals for the five years and the Z scores are also presented.

## Discussion

This study supports earlier observations that more males than females were buried in plague years in early modern London which suggests a higher mortality of males compared to females. Our results suggest that this observation may be extended generally to parishes situated within-the-walls over several plague years from 1563 to 1665 inclusive, although there is great variation between parishes and, sometimes, between years within the same parish. However, we also find a consistent but lesser bias towards male burials in non-plague years indicating that the greater mortality of males cannot wholly be attributed to plague. This ‘normal’ bias is likely to be the result of the underlying demography of London where there may have been a preponderance of males in the population due, in part, to the capacity of the metropolis to attract young men for the purpose of apprenticeship [28, 29].

When ‘adults’, ‘servants’ and ‘children’ were considered separately, contrasting effects of plague were revealed. We found no evidence of a change in the burial sex ratio for servants in plague years compared to non-plague years. On the other hand, ‘adults’ exhibited a significant increase in male burials compared to females, especially for the plague year 1603. Our analysis of baptismal data suggests that this could partly be attributed to the migration out of London by pregnant women as evidenced by the reduction in baptisms in the latter half of the plague years 1603, 1625 and 1665. Arguably, this diminution of birth rate might, in some way, be a consequence of a prior reduction in fertility/conceptions. However, the births recorded in the latter part of plague years must relate to conceptions occurring between early autumn of the previous non-plague year and early spring of the plague year yet outbreaks were typically seasonal and did not commence with full force until the late summer of the plague year. The possibility that it could be attributed to increased death in childbirth can also be discounted since this would have increased burials of women rather than have decreased them. Overall, these findings confirm John Graunt’s observations of a reduction in the rate of baptisms rate at the height of the plague in 1603 and indicate that this trend also seems to continue in the plague years 1625 and 1665.

An investigation of households revealed that significantly more husbands than wives were represented in the burial records in the plague years of 1603, 1625 and 1665. Yet again, this effect seemed to be most pronounced in 1603. Nevertheless, this finding cannot be accepted unreservedly since it relies on the assumption that each household is headed by a male-female partnership leading to an expectation to find equal numbers of males and female burials in our analysis. Undeniably, early modern society was shaped by the institution of marriage which was promoted and controlled by the church and courts thus we would expect that most household would be based upon the nuclear family [30]. Furthermore, the incidence of households led by widows or widowers is also likely to have been reduced by remarriage which was a relatively frequent event [31]. Nevertheless, there is evidence of households that depart from the nuclear family structure. For instance, in a sample of 40 households in the parish of St Mary, Colechurch in 1574, 31 were headed by a married couple, 2 by women and 7 by unmarried men [32]. In our study 75.5% of all household sampled recorded the death of at least one child thus implying that most households had, indeed, been based on the nuclear family structure. Thus, it seems unlikely that the strong bias towards male burials in this study can be explained by relatively small numbers of non-nuclear families.

For children, however, there was a clear bias towards the burial of boys in non-plague years compared to girls but, unexpectedly, in non-plague years the burial rates tended to approach parity. This is opposite to the trend observed in adults. This finding concurs with more limited observations by Finlay who compared the survival of boys and girls until the age of fifteen in four London parishes [3]. Finlay went further by suggesting that this bias was most obvious in poorer parishes although his sample size was too small to be statistically conclusive. The ratio of boys to girls in non-plague years is to be expected, given the greater vulnerability of male children over time compared to females in terms of perinatal causes and disease. That this ratio approaches parity in plague years requires explanation. It could be that girls were more vulnerable to plague in some way thus increasing their representation in the statistics of plague years. This could be because of some biological predisposition or a behavioural change. In the latter case one might speculate that older girls were called upon to carry out nursing activities, particularly if mothers departed and this might have made them more vulnerable through greater exposure to insect vectors or plague victims since it is well established that plague can be transmitted via fleas, ectoparasites and/or respiratory droplets [33]. Nevertheless, female servants were also placed in this position yet there is no evidence of a significant change in the sex ratio in this social category. An alternative hypothesis is that plague killed children indiscriminately and, due to the greatly enhanced death rates in plague years, the normal bias in death rate towards male children resulting from the usual range of pathogens circulating in the population would be hidden. In this respect our child mortality ratios (CMRs) ranged from 2.91 to 6.71 and Finlay calculated CMRs, ranging from 6-fold to 21-fold, for children in four London Parishes in 1593, 1603 and 1625 [3]. As far as we are aware, this is the first report that plague reduces the expected bias towards male deaths in children and it remains to be seen if this finding can be replicated for other medieval and early modern epidemics or if the phenomenon can be satisfactorily explained.

Our results seem to corroborate Finlay’s observation that there may have been something special about the year of 1603 although we note that 1625 was similar in some respects. In actual fact 1603 was a highly significant year politically, for it marked the death of Queen Elizabeth I and the accession of James I (James VI of Scotland) together with the union of the English and Scottish Crowns on the 24^th^ March. 1625 was also politically significant for it marked the death of James I and the accession of Charles I. The accession of James had encouraged a great influx of visitors to the capitol and thus the prospect of an extraordinary revival of trade as the contemporary observer Dekker [34] tells us:

“Now the thriftie Citizen casts beyond the Moone, and seeing the golden age returned into the world againe, resolves to worship no Saint but money. Trades that lay dead and rotten, and were in all mens opinion utterly dambd, started out of their trance, as though they had drunke of Aqua Caelestis, or Unicornes horne, and swore to fall to their olde occupation.”

Thus, it is possible that men of business might have encouraged their wives to depart from London whilst they remained to take advantage of the increase in trade made available by the coronation and influx of visitors. A similar process may have taken place in the plague year of 1625 following the death of James I which occurred on the 27^th^ March of that year. Charles I married his wife Henrietta Maria by proxy at Notre-Dame on 1^st^ May and met her at Dover on 13^th^ June. On 16^th^ June the couple entered London and Parliament assembled on the 18^th^ June having been prorogued on account of disease since the previous November [35]. The impact of these events, and thus the dilemma faced by men of business, is recalled thus by John Chamberlin in a contemporaneous letter to Sir Dudley Carlton [36]:

“Though the sickness increase shrewdly upon us so that this week died 640 in all, of the plague 239, and though this terme be abridged…yet we cannot find it in our hearts to leave this towne as long as here is such doings by reason of the Quenes arrival, and the sitting of parlement.”

We nevertheless advocate caution, since whilst most conclusions in this paper are supported by data and robust statistical analysis, we are only able to support this conjecture by circumstantial evidence and a single anecdote. Further studies are required to establish if it has any relevance to our principle findings.

Whether or not the reluctance of men to join their wives in escaping from the plague was significant, this study shows that the effect of plague on the relative frequency of death (as measured by burial records) of males and females in early modern London parishes is complex, involving both demographical and biological factors. However, the parishes observed in this study were mostly concentrated in the richer centre of the City of London which has an area no more than 1.12 sq. mi. (2.90 km^2^) and it would be unwise to extrapolate unguardedly to poorer parishes outside the walls, rural areas and, for that matter, to other countries where the circumstances and demographic constitution of the population might be entirely different. However, it does suggest that explanations need to reflect the demography and behaviour of the population under study as well as biological factors.
